# Is body size important? Seasonal changes in morphology in two grass-feeding *Abacarus* mites

**DOI:** 10.1007/s10493-017-0159-1

**Published:** 2017-07-27

**Authors:** Alicja Laska, Brian G. Rector, Lechosław Kuczyński, Anna Skoracka

**Affiliations:** 1 Population Ecology Lab, Institute of Environmental Biology, Faculty of Biology, Adam Mickiewicz University, Poznań, Umultowska 89, 61–614 Poznań, Poland; 2 Great Basin Rangelands Research Unit, 920 Valley Road, Reno, NV 89512 USA

**Keywords:** Eriophyoidea, Herbivores, Invertebrates, Phenotype

## Abstract

Overwintering strategies in herbivorous mites (Acariformes: Eriophyoidea) are poorly understood. A study of two *Abacarus* spp. was conducted to compare body size parameters of adult females in different seasons. Mites of *Abacarus* n. sp. (under description) and *A. lolli* were sampled from *Bromopsis inermis* and *Lolium perenne*, respectively, in April, September and December of 2001 in Poznań, Poland; 21 morphological traits were measured for each specimen. A principal component analysis revealed significant differences in body size parameters between collection dates, with larger females collected in December in both species. Larger body size in winter is consistent with the hypothesis that mites of these species, for which deutogyny has not been observed, undergo physiological changes such as accumulation of nutritional reserves, that enable them to withstand adverse environmental conditions. Larger body size has also been shown in other invertebrates to reduce heat loss in cold conditions. Filling gaps in the current knowledge of eriophyoid overwintering strategies, whether in the presence or absence of deutogyny, will contribute to both basic and applied future studies of this important arthropod group.

**Electronic supplementary material:**

The online version of this article (doi:10.1007/s10493-017-0159-1) contains supplementary material, which is available to authorized users.

## Introduction

In temperate climates, where annual temperature amplitude can exceed 40 °C, overwintering poses a significant challenge to ectothermic organisms. To successfully colonize such environments, they must be able to withstand harsh conditions, including extremes in temperature and humidity, increased predation risk and decreased food availability (Bagøien et al. 2001; Danks 2007; Luypaert et al. 2015). Overwintering mortality as high as 100% has been observed in field populations of some invertebrates (Pfrimmer and Merkl 1981; Régnière and Duval 1998). In order to cope with harsh winter conditions invertebrates may exhibit behavioral, physiological, biochemical or morphological adaptations (Danks 2004a). One such behavioral strategy is to avoid harsh conditions through migration (Irons et al. 1993) although this is only viable for organisms that are capable of traveling a sufficient distance away from the harsh conditions and returning of their own volition. Alternatively, ectotherms may adapt to overwintering in place (Danks 2007) through behavioral changes such as dormancy (Caceres 1997), building a hibernaculum (i.e. a protective winter cocoon; Régnière and Duval 1998), or seeking shelter, e.g., under tree bark, to protect against cold and moisture loss during the winter (Danks 2007; Lee et al. 2014).

Biochemical strategies may be employed, such as producing cryoprotectants to protect tissues from freezing damage (Storey and Storey 1991). These can be divided into two general groups: antifreeze compounds such as glycerol (and other polyhydric alcohols), that lower the super-cooling point (Salt 1959); and anti-nucleating agents that inhibit ice-crystallization (Zachariassen and Hammel 1976; Holmstrup and Zachariassen 1996). Invertebrates may also employ physiological adaptations, such as accumulating resources in tissues to avoid starvation. Such a strategy may result in increased body mass (Convey 1994).

Another strategy for coping with extreme conditions is to modify developmental stages or rates. This strategy may include either accelerated or delayed development in order to overwinter in the most resistant stage or the development of a dedicated overwintering state, such as diapause (Danks 2007). Diapause typically involves a reduction in metabolic activity and an interruption in development, allowing the organism to conserve bodily resources until more favorable environmental conditions return (Danks 2004b). In eriophyoid mites species living in temperate regions, overwintering females known as deutogynes or “winter forms” or “secondary forms” are able to survive cold conditions. They appear in late summer and pick up spermatophores, then move into sheltered crevices on twigs, under bud scales or around lateral buds where they hibernate. They overwinter as inseminated females, but can lay eggs only when they go through a period of winter cold. In spring they emerge to lay eggs, from which protogyne females and males develop (Manson and Oldfield 1996). However, it should be mentioned that deutogyne females also occur in species outside temperate regions. In general, deutogyny promotes survival through adverse conditions and this form of females is specialized to withstand extremely cold or hot conditions (Manson and Oldfield 1996).

Deutogyne forms usually differ morphologically from protogyne forms, for example by having reduced or suppressed microtuberculation, different microtubercle shapes, a loss of dorsal strial lobes from the integument, narrower tergites, or less ornamentation (e.g., lacking ridges, furrows or protuberances that occur in the protogyne; Jeppson et al. 1975; Manson and Oldfield 1996). Differences in body size have been recorded between protogyne and deutogyne forms of eriophyoid mites, with two opposing patterns having been observed. Britto et al. (2008) described deutogyne females of *Aceria inusitata* that were fusiform in their shape and much larger when compared to vermiform protogyne females. In this tropical species deutogynes also have an adaptive behavior, in that they build nests that are necessary for protogyne survival (Britto et al. 2008). Deutogynes of *Tegolophus celtis* infesting Chinese hackberry (*Celtis sinensis*) were much larger than the protogyne form; they also differed in colour (red vs. white) (Guo et al. 2015). Other authors, however, found that protogyne females were larger (Somsen 1966; Jeppson et al. 1975; Druciarek et al. 2016; Liu et al. 2016). Smaller size in deutogyne females of *Aceria pallida* is adaptive for phoresy and overwintering in association with the insect *Bactericera globica* (Liu et al. 2016), whereas smaller deutogyne females in grass-associated *Aceria* species are believed to be adapted for dispersal both before and after the overwintering period (Somsen 1966; Jeppson et al. 1975; Manson and Oldfield 1996). Sometimes differentiation between protogyne and deutogyne forms is difficult, as in the case of *Aceria anthocoptes* and *A. leonthodontis*, both associated with *Cirsium arvense*; in fact they appear to be protogyne and deutogyne forms, respectively, of a single species (Petanovic et al. 1997). Little is known about the specific physiological adaptations that presumably accompany morphological alterations recognized as deutogyny. It has been suggested that modification of microtubercles on the opisthosoma may render the cuticule more resistant to water loss (Manson and Oldfield 1996).

Knowledge of the overwintering strategies of the majority of eriophyoid species in which deutogynes are unknown is scarce. The survival of eriophyoid mites, which are obligate herbivores, living in temperate regions is dependent on the availability of suitable host plants and should be coupled with the ability to withstand cold temperatures, given their inability to migrate. In some species in the genera *Eriophyes, Phytoptus*, and *Aceria*, mites move from leaves into leaf buds before winter where they hibernate until the following spring (Manson and Oldfield 1996). Species inhabiting forbs or grasses may overwinter within protected recesses of their host plants. For example, the wheat curl mite, *Aceria tosichella*, shelters in part of its grass hosts that remain through the winter; all stages can survive the coldest temperatures (Slykhuis 1955; Nault and Styer 1969). Also, in the *Abacarus hystrix* species complex, mites overwinter in the crowns of grass host plants; reproductive and developmental activity is reduced but no diapauses or quiescent stage has been observed (Frost and Ridland 1996). Given that deutogyne females have not been observed in grass-feeding *Abacarus* species, comparable overwintering strategies would be expected, such as the synthesis of cryoprotectants or sequestration of nutrients that would be crucial during an extended period without feeding. Such strategies may be reflected in the morphology of the mites but to date no examples of morphological changes (including body size changes) in overwintering protogyne females of eriophyoid mites have been reported.

The purpose of this study was to compare seasonal body morphology in two grass-feeding *Abacarus* species for which deutogyny has never been observed: *A. lolii* collected from *Lolium perenne* and *Abacarus* n. sp. (a new species currently under description) that was collected from *Bromopsis inermis* and is distinguished from *A. lolii* on the basis of morphology, host plant, and sequence polymorphisms in two common marker genes (Skoracka et al. 2002, 2007; Skoracka and Kuczyński 2006a, b; Skoracka 2009; Skoracka and Dabert 2010). We hypothesized that protogyne females of these two species would have a larger overall body size in winter than in either spring or summer as a result of nutrients accumulated in tissues to withstand the cold season.

## Materials and methods

Plant samples of *L. perenne* and *B. inermis* were collected in Poznań, Poland, on 30 April, 23 September, and 27 December 2001, in Cytadela Park (52°25′18′′N, 16°56′10′′E). As in Poland aboveground grass plants can remain alive during winter, it was possible to collect mites in this season; mites were alive and all stages were found. Immediately after collection, plants were transported to the laboratory, where they were directly inspected for the presence of mites under a stereo-microscope. Mite specimens were transferred to slides using an eyelash glued to a dissecting needle and mounted in modified Berlese medium (Monfreda et al. 2010) in dorsoventral orientation. Slides were examined using a phase-contrast microscope (Olympus BX41) and all mite specimens were morphologically identified as *Abacarus hystrix* sensu lato, which has been revealed to be a species complex (Skoracka and Dabert 2010) that now includes *A. hystrix* sensu stricto, *A. lolli* (Skoracka 2009) and *Abacarus* n. sp. (under description). In this study, all specimens collected from *L. perenne* were identified as *A. lolii* and those collected from *B. inermis* as *Abacarus* n. sp. Each sample consisted of 30 grass shoots, from which 30 females were randomly selected. Twenty-one morphological traits were measured for each specimen: length of body, length of prodorsal shield, width of prodorsal shield, length of scapular setae *sc*, distance between tubercles of scapular setae *sc*, length of setae *c2*, length of setae *d*, length of setae *e*, length of setae *f*, length of female genitalia, width of female genitalia, length of genital setae *3a*, distance between genital tubercles, distance between 1st tubercles of coxa I, distance between 2nd tubercles of coxa *II*, distance between tubercles of coxa *III*, length of coxal setae *2a*, length of *I* tibia, length of *I* tarsus, length of *II* tibia, and length of *II* tarsus.

A principal component analysis (PCA) was applied to reduce dimensionality in the raw morphometric data. A variance–covariance matrix was used, as all original measurements were made on the same metric scale. Then, multivariate analysis of variance (MANOVA) was used to test the effects of season and mite species on the morphological traits, as well as their interaction, expressed by means of the most important principal components. Finally, a one-way ANOVA was applied to test the same effects using individual principal components. For each component considered, within-species comparisons were made with regard to the month of mite collection. As this procedure involves multiple comparisons, we controlled the error rate by applying a method proposed by Bretz et al. (2011). For all computations, R version 3.3 was used (R Development Core Team 2016).

## Results

Two principal components were extracted that accounted for 89.7% of the total variance in the morphometric data (Table 1). The loadings of the first axis were all positive and thus can be associated with the overall body size. The second principal component was positively correlated with body length and negatively correlated with all other traits. The higher the value of this component, the more elongated the animal, with shorter legs and setae, as well as a smaller prodorsal shield.

**Table 1:** Principal component analysis of the morphometric data, showing loadings of the first two PCA factors

Trait	Comp.1	Comp.2
Length of body	0.96	0.28
Length of prodorsal shield	0.06	−0.06
Width of prodorsal shield	0.07	−0.03
Length of scapular setae *sc*	0.06	−0.03
Distance between tubercles of setae *sc*	0.05	−0.06
Length of setae *c*2	0.15	−0.01
Length of setae *d*	0.14	−0.01
Length of setae *e*	0.11	−0.02
Length of setae *f*	0.04	−0.06
Length of female genitalia	0.02	−0.17
Width of female genitalia	0.02	−0.16
Length of genital setae 3*a*	0.09	−0.03
Distance between genital tubercles	0.02	−0.12
Distance between 1st tubercles of coxa *I*	0.02	−0.31
Distance between 2nd tubercles of coxa *II*	0.02	−0.30
Distance between tubercles of coxa *III*	0.03	−0.07
Length of setae 2*a*	0.07	−0.03
Length of *I* tibia	0.02	−0.17
Length of *I* tarsus	0.01	−0.51
Length of *II* tibia	0.01	−0.32
Length of *II* tarsus	0.01	−0.49
%Variance explained (cumulative)	70.8	89.7

**Fig. 1:**
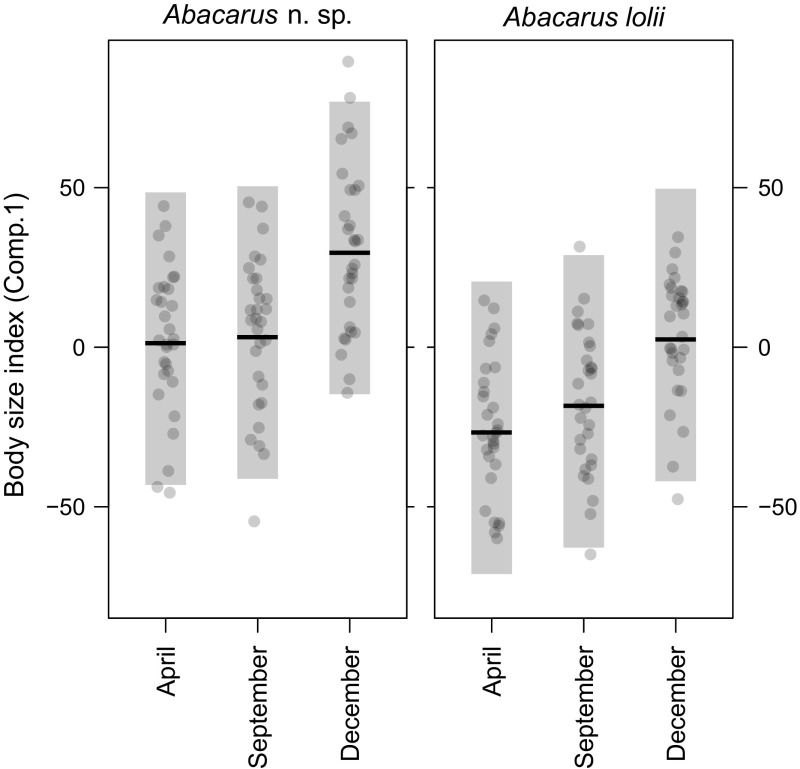
Means (*horizontal lines*), 95% prediction intervals (*shaded regions*), and raw values (*points*) for the first principal component (*body size index*) for *Abacarus* n. sp. and *Abacarus lolii* collected in April, September and December. *Body size index* in both species was significantly greater in December than in either April or September, whereas the differences between April and September were insignificant (*p* values for contrasts are given in the Table 3)

**Fig. 2:**
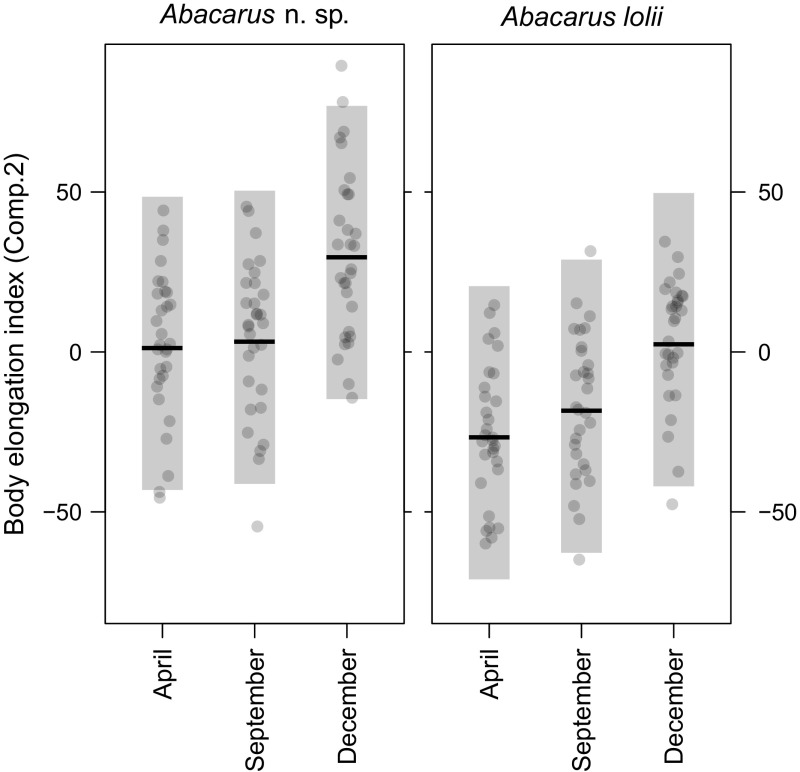
Means (*horizontal lines*), 95% prediction intervals (*shaded regions*), and raw values (*points*) for the second principal component (*body elongation index*) for *Abacarus* n. sp. and *Abacarus lolii* collected in April, September and December. Values of this component increase throughout the year for *Abacarus* n. sp., while whereas the morphology of *A. lolii* differed significantly only between April and September (*p* values for respective contrasts are given in the Table 4)

MANOVA revealed significant effects of both season and species, as well as their interaction on mite morphology (Table 2). The one-way ANOVA applied to the first principal component revealed significant effects of both season (F_2, 174_ = 27.1, *p* < 0.0001) and species (F_1, 174_ = 56.5, *p* < 0.0001) on mite body size. However, the interaction of the two factors was not significant (F_2, 174_ = 0.35, *p* = 0.7030), meaning that this pattern was consistent for both studied mite species (Table S1, Online Resource 1). Analysis of contrasts showed that body size was greater in December than in either April or September, while the differences between April and September were insignificant (Table 3; Fig. 1). The one-way ANOVA applied to the second principal component was significant for the main effects: season (F_2, 174_ = 9.82, *p* < 0.0001) and species (F_1, 174_ = 392.80, *p* < 0.0001). In this case the interaction was significant (F_2, 174_ = 26.89, *p* < 0.0001; Table S2, Online Resource 2), indicating different seasonal patterns between species. One species (*Abacarus* n. sp.) retained its body length vs. setae length ratio throughout the year, with the value of principal component 2 increasing from April through September to December. The morphology of the second species (*A. lolii*) did not change during the year in the manner observed for *Abacarus* n. sp. Measured *A. lolii* specimens differed significantly only between April and September (Table 4; Fig. 2).

**Table 2:** MANOVA for PCA components 1 and 2 (for both species)

Factor	*df*	Pillai’s trace	F	Numerator* df*	Denominator* df*	*P*
Month	2	0.2436	12.1	4	348	<0.0001
Host	1	0.8755	608.2	2	173	<0.0001
Month × host	2	0.3846	20.7	4	348	<0.0001
Residuals	174					

MANOVA exposes significant effects of season, species and their interaction on mite morphology

**Table 3:** Analysis of contrasts for the first principal component

Linear hypotheses	Contrast	SE	t	*p*
Apr.BI–Sep.BI = 0	−1.91	5.90	−0.32	0.9968
Apr.BI–Dec.BI = 0	−28.40	5.90	−4.82	<0.0001
Sep.BI–Dec.BI = 0	−26.49	5.90	−4.49	<0.0001
Apr.LP–Sep.LP = 0	−8.30	5.90	−1.41	0.5625
Apr.LP–Dec.LP = 0	−29.10	5.90	−4.93	<0.0001
Sep.LP–Dec.LP = 0	−20.80	5.90	−3.53	0.0031

Contrasts were calculated for groups defined by the month of mite collection (Apr, April; Sep, September; Dec, December) and mite species (BI, *Abacarus* n. sp.; LP, *Abacarus lolii*)

**Table 4:** Analysis of contrasts for the second principal component

Linear hypotheses	Contrast	SE	t	*p*
Apr.BI–Sep.BI = 0	−7.68	2.03	−3.78	0.0012
Apr.BI–Dec.BI = 0	−15.75	2.03	−7.76	<0.0001
Sep.BI–Dec.BI = 0	−8.07	2.03	−3.98	<0.0001
Apr.LP–Sep.LP = 0	7.30	2.03	3.60	0.0024
Apr.LP–Dec.LP = 0	4.55	2.03	2.24	0.1296
Sep.LP–Dec.LP = 0	−2.75	2.03	−1.35	0.5983

Contrasts were calculated for groups defined by the month of mite collection (Apr, April; Sep, September; Dec, December) and mite species (BI, *Abacarus* n. sp.; LP, *Abacarus lolii*)

## Discussion

The morphology of the two *Abacarus* species in this study changed according to the season; specifically, the mites had increased body size in winter in comparison to spring and late summer, supporting the stated hypothesis. This pattern was consistent in the two species, which share a similar seasonal cycle but differ with respect to host-species (Skoracka and Dabert 2010). Similarly, Kuo et al. (2013) found that specimens of another *Abacarus* species, *A. panticis*, infesting Yushan cane in Taiwan, were larger in lower temperatures, although this was associated with higher elevations rather than seasonality. Seasonal variation in body size in species with no diapause adaptation has also been described in several different invertebrate groups, mostly in insects, including some stoneflies (Haro et al. 1994), black flies (Colbo and Porter 1979; Baba 1992), *Drosophila* spp. (Tantawy 1964; Kari and Huey 2000), mosquitoes (Yuval et al. 1993), tsetse flies (Rogers and Randolph 1991), beetles (Ernsting and Isaaks 1997), butterflies (Rodrigues et al. 2004), parasitoid wasps (Sequeira and MacKauer 1993), and bees (Alcock et al. 2005; Classen et al. 2017). The majority of these studies indicated that temperature changes have a significant influence on body size and that body size tends to be largest at the lowest temperatures (Chown and Gaston 2010).

The observed phenomenon could be interpreted as an adaptation for overwintering since enlarged body size may be a result of storing reserves, especially lipids in the fat body (Danks 2007). This strategy may improve resistance to starvation (e.g., Pincheira-Donoso et al. 2008). For example, Cuber et al. (2016) observed that the fatty acid profile of *Ixodes ricinus* ticks varied significantly from season to season, and their content tended to increase at lower temperatures. The ameronothrid mite *Alaskozetes antarcticus* exhibits a similar pattern of rapid growth during a brief summer period, resulting in large bodies at the beginning of winter (Convey 1994). Increased body size in the *Abacarus* spp., shown in this study could reflect such storage of energy reserves and these physiological adaptations merit measurement in future studies.

The second outcome from this study indicated that other species-specific morphological traits, viz. body elongation versus legs, setae and prodorsal shield length, may also be linked to environmental factors. *Abacarus* n. sp., specimens were the longest in winter while at the same time their legs and ventral and lateral setae were shortest; by contrast, their setae and legs were the longest in April. *Abacarus lolii* specimens had the longest legs and setae in September and the shortest in April. Setae in eriophyoid mites may play a role during aerial dispersal (Krantz 1973), which is the major mode of eriophyoid spread over long distances (Michalska et al. 2010). Walking is another important way of moving within a plant or in habitats where branches or leaves of suitable plants contact one another (Michalska et al. 2010). Neither wind dispersal nor ambulatory movement are expected to take place in winter due to unfavorable climatic conditions and a general lack of fresh host plants, whereas in spring and summer eriophyoid wind dispersal is common (Nault and Styer 1969). The morphological patterns observed in the *Abacarus* species studied here may reflect a plasticity of morphological traits depending on seasonal factors. Alternatively, these results may suggest that these two species disperse in different seasons, perhaps in association with differing phenologies between their host species. *L. perenne* is a low-growing, tufted plant. It occurs mostly in meadows and pastures, and it flowers from May to September. *Bromopsis inermis* is a long-lived, rhizomatous grass, commonly producing a dense sod. It occurs in ruderal places and flowers in June and July (Szafer et al. 1969). More detailed study of seasonal differences in the two grass host species may shed light on such associations.

To the best of our knowledge, the influence of seasonal factors on eriophyoid mite morphology has not been studied to date. Earlier studies investigating eriophyoid morphological changes focused on host- or spatially-related variation, which has most frequently been attributed to partial or total separation of gene pools due to host-associated differentiation or geographic speciation (Skoracka et al. 2002, 2014; Lewandowski et al. 2014; Li et al. 2014; Vidović et al. 2014; Navia et al. 2015; Živković et al. 2017). The precise stimuli that ultimately result in increased winter body size in the two *Abacarus* spp., studied here are not known. While invertebrates with larger bodies may resist cold temperatures better than smaller ones, as discussed above, it is not known whether the changes observed in this study arose due to the falling temperatures themselves or other seasonal factors (e.g., changing photoperiod) or due to changes that the mites detect in their host plants. Elucidation of such questions should be relatively straightforward through laboratory experiments under controlled conditions.

In conclusion, enlarged body size as an adaptation to low temperature and adverse environmental conditions is a common strategy in ectothermic organisms that is also reflected in seasonal changes in physiology, behavior and metabolism ratio (Danks 2007; Chown and Gaston 2010). Body size may be related to overwintering in grass-feeding *Abacarus* mites based on the data from the two species studied here. Much knowledge about seasonal variability and diversity of morphology in eriophyoid mites is still lacking. It is not known, for example, how body mass changes during the changing seasons or whether it is associated with accumulated nutrients or other physiological changes. The information gained from this study can be used to develop hypotheses regarding physiological adaptation to overwintering in the studied species. Results of such research can be useful in the development of effective plant protection and management strategies for related pest species. Detailed study of the life cycle of *Abacarus* under controlled conditions to corroborate changes in morphology and physiology with changes in ambient temperature can offer clues to their ability to avoid winter mortality and increase population growth with the return of favorable conditions.

## Electronic supplementary material

Below is the link to the electronic supplementary material.

Supplementary material 1 (DOCX 17 kb)

Supplementary material 2 (DOCX 16 kb)
