# Supplementary material for: Is body size important? Seasonal changes in morphology in two grass-feeding Abacarus mites
Source: Exp Appl Acarol. 2017 Jul 27;72(4):317–28. doi: 10.1007/s10493-017-0159-1 (PMC5583266; doi:10.1007/s10493-017-0159-1)
Supplement: Supplementary file 2 — Supplementary material 2 (DOCX 16 kb) [file 10493_2017_159_MOESM2_ESM.docx]

Is body size important? Seasonal changes in morphology in two grass-feeding *Abacarus* mites.

Experimental and Applied Acarology

Alicja Laska^1*^, Brian G. Rector^2^, Lechosław Kuczyński^1^, Anna Skoracka^1^

^1^Population Ecology Lab, Institute of Environmental Biology, Faculty of Biology, Adam Mickiewicz University, Poznań, Umultowska 89, 61–614 Poznań, Poland

^2^Great Basin Rangelands Research Unit, 920 Valley Road, Reno, NV 89512, USA

*corresponding author: Alicja Laska, e-mail address: [alicja.laska@amu.edu.pl](mailto:alicja.laska@amu.edu.pl); telephone number: 0048 618295749

**Table S2** One-Way ANOVA applied for comp.2 calculated for both species shows significant effect of all examined factors – season, species and interaction of them

| Factor | Df | Sum Sq | Mean Sq | F value | Pr (>F) |
| --- | --- | --- | --- | --- | --- |
| Month | 2 | 1214 | 607 | 9.82 | <0.0001 |
| Host | 1 | 24279 | 24279 | 392.80 | <0.0001 |
| Month:host | 2 | 3324 | 1662 | 26.89 | <0.0001 |
| Residuals | 174 | 10755 | 62 |  |  |
